# Inequalities in urban air pollution in sub-Saharan Africa: an empirical modeling of ambient NO and NO_2_ concentrations in Accra, Ghana

**DOI:** 10.1088/1748-9326/ad2892

**Published:** 2024-02-27

**Authors:** Jiayuan Wang, Abosede S Alli, Sierra N Clark, Majid Ezzati, Michael Brauer, Allison F Hughes, James Nimo, Josephine Bedford Moses, Solomon Baah, Ricky Nathvani, Vishwanath D, Samuel Agyei-Mensah, Jill Baumgartner, James E Bennett, Raphael E Arku

**Affiliations:** 1 Department of Environmental Health Sciences, School of Public Health and Health Sciences, University of Massachusetts, Amherst, MA, United States of America; 2 Department of Epidemiology and Biostatistics, School of Public Health, Imperial College London, London, United Kingdom; 3 MRC Centre for Environment and Health, School of Public Health, Imperial College London, London, United Kingdom; 4 Regional Institute for Population Studies, University of Ghana, Accra, Ghana; 5 Abdul Latif Jameel Institute for Disease and Emergency Analytics, Imperial College London, London, United Kingdom; 6 School of Population and Public Health, The University of British Columbia, Vancouver, Canada; 7 Department of Physics, University of Ghana, Accra, Ghana; 8 Department of Geography and Resource Development, University of Ghana, Accra, Ghana; 9 Institute for Health and Social Policy, McGill University, Montreal, Canada; 10 Department of Epidemiology, Biostatistics, and Occupational Health, McGill University, Montreal, Canada; 11 Department of Civil and Environmental Engineering, Imperial College London, London, United Kingdom

**Keywords:** air pollution, nitrogen dioxide (NO_2_), nitrogen oxides (NO*x*), sub-Saharan Africa, Ghana, air pollution inequality, land use regression

## Abstract

Road traffic has become the leading source of air pollution in fast-growing sub-Saharan African cities. Yet, there is a dearth of robust city-wide data for understanding space-time variations and inequalities in combustion related emissions and exposures. We combined nitrogen dioxide (NO_2_) and nitric oxide (NO) measurement data from 134 locations in the Greater Accra Metropolitan Area (GAMA), with geographical, meteorological, and population factors in spatio-temporal mixed effects models to predict NO_2_ and NO concentrations at fine spatial (50 m) and temporal (weekly) resolution over the entire GAMA. Model performance was evaluated with 10-fold cross-validation (CV), and predictions were summarized as annual and seasonal (dusty [Harmattan] and rainy [non-Harmattan]) mean concentrations. The predictions were used to examine population distributions of, and socioeconomic inequalities in, exposure at the census enumeration area (EA) level. The models explained 88% and 79% of the spatiotemporal variability in NO_2_ and NO concentrations, respectively. The mean predicted annual, non-Harmattan and Harmattan NO_2_ levels were 37 (range: 1–189), 28 (range: 1–170) and 50 (range: 1–195) *µ*g m^−3^, respectively. Unlike NO_2_, NO concentrations were highest in the non-Harmattan season (41 [range: 31–521] *µ*g m^−3^). Road traffic was the dominant factor for both pollutants, but NO_2_ had higher spatial heterogeneity than NO. For both pollutants, the levels were substantially higher in the city core, where the entire population (100%) was exposed to annual NO_2_ levels exceeding the World Health Organization (WHO) guideline of 10 *µ*g m^−3^. Significant disparities in NO_2_ concentrations existed across socioeconomic gradients, with residents in the poorest communities exposed to levels about 15 *µ*g m^−3^ higher compared with the wealthiest (*p* < 0.001). The results showed the important role of road traffic emissions in air pollution concentrations in the GAMA, which has major implications for the health of the city’s poorest residents. These data could support climate and health impact assessments as well as policy evaluations in the city.

## 1. Introduction

Cities in sub-Saharan Africa (SSA) are in an economic transition and undergoing significant expansion. With such rapid growth, SSA cities are experiencing high levels of air pollution from diverse sources [1]. The growth is also changing the air pollution mixture and the relative roles of the major emission sources. Recent studies suggest that the dominant emission source of urban air pollution in SSA may be shifting from household biomass burning [2, 3] to road traffic [4, 5]. Consequently, while the concentrations of fine particulate matter pollution (PM_2.5_) are showing signs of plateauing [4], several studies are reporting steady increases in oxides of nitrogen (NO*_x_*) pollution [5–8], which are markers of traffic emissions in cities. Increasing formal and informal industrial activities as well as household and commercial use of diesel generators are also common in SSA cities and contribute substantially to ambient NO*_x_* levels. The distribution of these sources in relation to land use and socioeconomic factors influences the spatial patterns of NO*_x_* pollution in local communities [4, 5, 9–14]. For cities across the West African sub-region, seasonal changes in regional meteorological parameters (e.g. mixing layer depth, incident solar radiation and water vapor mixing ratio) during the dry and dusty Harmattan may also amplify NO*_x_* concentrations from local emissions during this period [5, 11, 12]. Yet, there is a dearth of long-term monitoring data for understanding trends, space-time variations and inequalities in combustion related emissions and exposures at city-scale in SSA, one of the world’s fastest urbanizing regions.

Nitrogen dioxide (NO_2_), the largest component of NO*_x_*, is associated with adverse health impacts such as inflammation of the airways and impaired lung function [15, 16]. Along with NO*_x_*, NO_2_ reacts with other chemicals in the air to form PM and ozone (O_3_). These photochemical reactions can also produce adverse impacts on the environment (e.g. formation of haze, smog, and acid rain). As such, national governments and international agencies have set health-based guidelines to reduce NO*_x_* emissions. Furthermore, NO_2_ is regularly monitored in cities in high-income countries. This is not the case in most sprawling cities in SSA, though the vehicle fleets contain high volumes of older, more polluting vehicles. Furthermore, because urban growth in SSA is largely unplanned, places with quality and healthy living environments are unequally distributed within cities. Although there are global satellite data on NO_2_ pollution covering the region [17], they do not capture the high within-city variability that characterizes localized emissions and sources in the context of SSA. To create accountability towards equitable urban living environments, local fine-scale data are needed for regulatory purposes as well as to identify and support deprived populations and communities. Such data are equally essential for health and climate impact assessments at the local community level in an exposure setting that is quite different from those in high-income country cities [18, 19]. In particular, NO*_x_* data, when combined with increasing data on PM_2.5_ and black carbon (BC), will deepen our understanding of the shifting emission sources that is happening in SSA cities that are in economic transition. The data will also enable SSA cities to design and implement integrated air quality management schemes to address growing urban air pollution problems in the region. Additionally, NO_2_ emissions serve as a general proxy for co-emitted pollutants (e.g. carbon monoxides and heavy metals) during fossil fuel combustion. Hence, knowledge of the patterns and concentrations of NO_2_ gives added information from the environmental justice perspective.

Previously, we described the levels and patterns of nitric oxide (NO) and NO_2_ pollution using year-long measurement data on NO*_x_* (*n* = 428 weekly samples) and NO_2_ (*n* = 472 weekly samples) from 134 monitoring sites in Accra, Ghana [5]. In this paper, we leveraged the measurement data to develop empirical (so called ‘land use regression’) models to map ambient NO and NO_2_ concentrations at fine spatiotemporal scales (weekly at 50 m) over the entire city. Model predictions were used to derive population exposure distributions and as well to investigate socioeconomic disparities in exposure across the metropolis. We are aware of only two small studies that mapped NO_2_/NO in SSA, but none in a large metropolis like Accra [20, 21].

## 2. Methods

### 2.1. Study area

Accra is one of the largest and fastest-growing metropolises in West Africa. Our study was conducted in the Greater Accra Metropolitan Area (GAMA), the capital of Ghana and home to an estimated six million residents [22]. GAMAs 1500 km^2^ area contains multiple administrative districts, including the city core and most populous Accra Metropolitan Area (AMA); and the Tema Municipal Area (TMA), the industrial hub and seaport situated east of AMA. Daily commute in the GAMA is characterized by heavy traffic congestions, with cars and ‘trotro’ (minibuses for public transport) alongside pedestrians [23]. There is limited formal bus transit and train services. Recently, there is a growing number of subcompact cars being used as ride-shares such as Uber and Bolt, and the use of motorcycle-taxis (‘Okada’) is on the rise. To fill the insufficient energy access gap in this growing economy, household and commercial use of diesel generators is commonplace. These are all sources of NO*_x_* emissions in the city. Despite the economic and technological advancement, there still exists immense inequalities in income, housing, infrastructure, and services, which also pattern disparities in environmental pollution within the city. The GAMA experiences two major seasons: the dry and dusty Harmattan (November to February), where north-easterly trade winds blows in mineral dusts from the Sahara Desert during a stagnant local meteorology; and the wet/rainy season (May to October), generally dominated by local air pollution sources [4, 5, 11, 12].

### 2.2. Data sources

#### 2.2.1. NO_2_ and NO_x_ measurement

**Figure 1.**
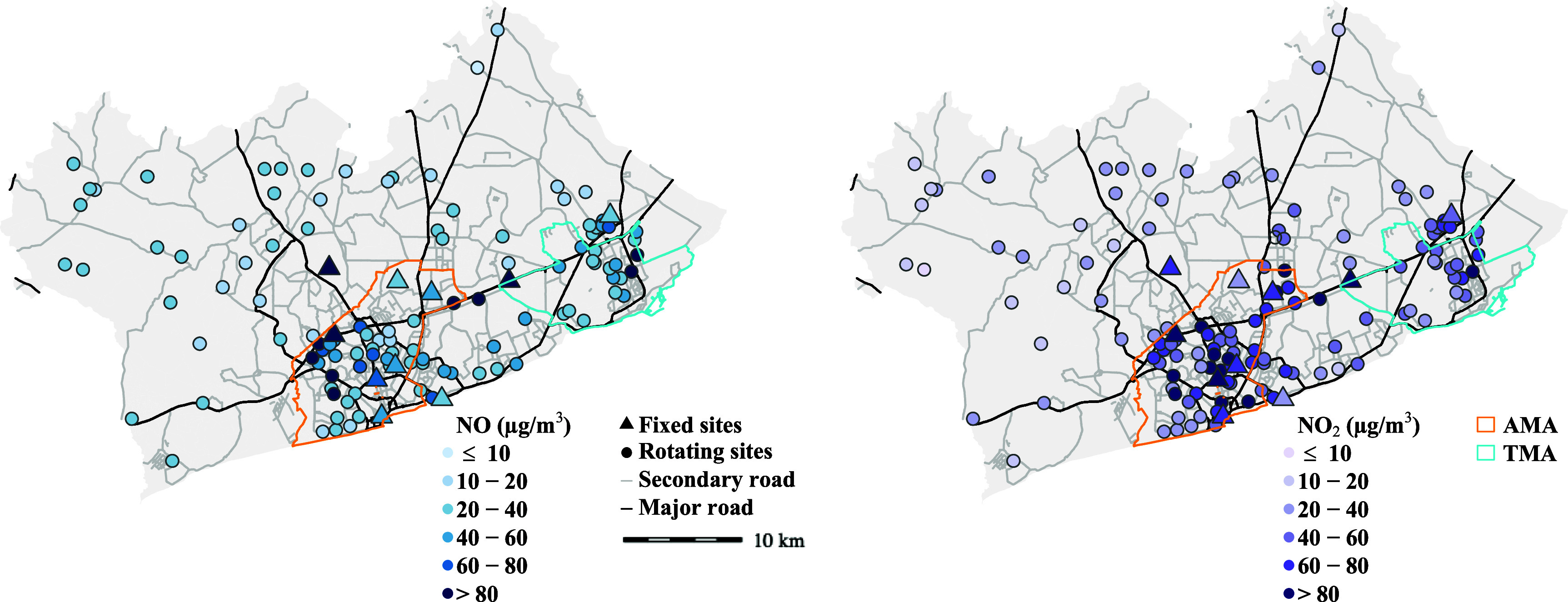
Map of the Greater Accra Metropolitan Area (GAMA) with locations of ‘*fixed*’ and ‘*rotating*’ sites with annual concentrations of (A) NO and (B) NO_2_. The colors of NO_2_ concentrations indicate comparison to the new World Health Organization (WHO) annual air quality guideline of 10 *μ*g m^−3^. The concentrations at the fixed sites represent annual mean values, and the rotating sites represent season-adjusted mean values (i.e. an estimated annual means). Major and secondary road network were from OpenStreetMap [27] (downloaded in 2019).

Detailed description of the measurement campaign and site selection can be found elsewhere [5]. Between April 2019 to June 2020, we collected weekly integrated NO_2_ and NO*_x_* samples at 134 unique sampling locations using Ogawa passive samplers. The 134 sites were chosen to cover diverse land use and socioeconomic status (SES) in the GAMA. As frequently used markers for traffic-related emission, we expected a high degree of inter- and intra-neighborhood variations in NO*_x_* pollution within GAMA. Thus, our sampling sites were over-represented in the more densely populated AMA relative to the rest of the GAMA, as a reflection of the population, land use and source features. Ten of the sites were sampled weekly for one year to capture longer-trend (‘*fixed sites’*) and 124 sites were sampled for one week each to allow for wider geographic coverage (‘*rotating sites’*) (figure 1). There were some missing data between March and April 2020, due to Covid-19 lockdown of Accra as well as mandatory quarantine for the field team through contact tracing. While Covid-19 partial lockdown affected emissions in the city briefly, but our analysis showed that the levels rapidly returned to pre-lockdown concentrations in the post-lockdown era^5^. Altogether, we collected a total of 428 and 472 weekly NO*_x_* and NO_2_ samples, respectively, comprising 281 NO_2_ and 251 NO*_x_* samples in the pre-Covid-19 lockdown, 19 pairs during Covid-19 lockdown, and 50 pairs in the post-Covid-19 lockdown periods. We collected field blank and duplicate samples at 20% of the rotating sites. All the raw data were blank-corrected, and the duplicates had good agreement (*R* ^2^ = 0.98 for NO*_x_*; and 0.95 for NO_2_) [5]. We did not collocate the Ogawa samplers against a reference NO*_x_*/NO_2_ monitor as they had been well-characterized in field settings with good agreements [24, 25], including in similar settings as ours [26]. We estimated NO from NO*_x_* as NO = NO*_x_* − NO_2_. The final weekly estimates were converted using temperature and relative humidity (RH) of that measurement week. We reported all results in *µ*g m^−3^ (1ppb NO_2_ ≈ 1.88 *µ*g m^−3^ and 1 ppb NO ≈ 1.23 *µ*g m^−3^, all the conversion factors between ppb and *µ*g m^−3^ were calculated based on weekly measured temperature and RH) for easy comparison with other studies and international health guidelines.

Full description of the NO and NO_2_ analysis and concentrations at the monitoring sites is available elsewhere [5]. In summary, NO and NO_2_ concentrations varied spatially (i.e. by land-use features) and temporally (by season), with annual mean for NO_2_ well above international health-based guidance (figure 1). The measured data were strongly associated with indicators of road traffic emissions and meteorological variables.

#### 2.2.2. Predictor variables

We gathered spatial and temporal predictor variables that reflect emissions and factors related to sources in the SSA urban environment (table 1). We first created four buffer sizes (50 m, 100 m, 200 m and 500 m) around each of the 134 sites. Within each buffer, we extracted multiple spatial predictor variables related to traffic (road network) emissions, land use, population, and human activities as described below in model selection. We used a road network shapefile from OpenStreetMap [27] (downloaded 2019) to estimate total length of major and secondary roads; distance from the monitor to the nearest major and secondary roads; and counts of bus/trotro stations/terminals. Total length of waterways (river, stream, canal and drain) were also estimated. We used Spot five imagery (2014) to calculate total area of land within each buffer that were characterized as commercial/business/industrial; high-density residential; low-density residential; and peri-urban background. Normalized difference vegetation index (NDVI) from Landsat-8 satellite imagery was used to characterize vegetation within each buffer size. Additionally, we used the 2010 national census data to compute population density and the share of households using biomass in each census EA, the smallest spatial administrative unit. Further, human activity data, including restaurants, bars, shops, schools, hospitals, churches, and mosques were retrieved from Google places in 2020. We could not find any reliable data on trash burning, fish smoking, generator use, traffic volume, and industrial emissions.

**Table 1.** Candidate predictor variables available for model selection.

Variables and categories	Unit	Buffer size (m)	Source
Traffic variables			OpenStreetMap (2019) [27]
Total length of major roads	m	50, 100, 200, 500	
Total length of secondary roads	m	50, 100, 200, 500	
Distance to the nearest major road	m	—	
Distance to the nearest secondaryroad	m	—	
Land use variables			World Bank [28] 20 m × 20 m
Commercial/business/industrial	m^2^	50, 100, 200, 500	
High-density residential	m^2^	50, 100, 200, 500	
Low/medium-density residential	m^2^	50, 100, 200, 500	
Peri-urban areas	m^2^	50, 100, 200, 500	
Normalized difference vegetationindex (NDVI)	—	50, 100, 200, 500	United States Geological Survey [29]—Landsat 8 imagery (30 m × 30 m)
Waterways (total length)	m	50, 100, 200, 500	OpenStreetMap [27, 2019]
Counts of building	N	50, 100, 200, 500	Maxar/Ecopia.ai [30, 2020]
Population			Ghana census (2010) data [31]
Biomass use	%	50, 100, 200, 500	
Population density	pop km^−2^	50, 100, 200, 500	
Human activities			Google Places (retrieved in 2020)
Number of restaurants	N	50, 100, 200, 500	
Number of schools	N	50, 100, 200, 500	
Presence of bars	N	50, 100, 200, 500	
Presence of shops	N	50, 100, 200, 500	
Meteorological variables			
Temperature	˚C	—	Kestrel weather meters
Relative humidity	%	—	Kestrel weather meters
Wind speed	m s^−1^	—	Kestrel weather meters
Mixing layer depth	m	—	HYSPLITE model [32]
Solar radiation	W km^−2^	—	HYSPLITE model [32]
Water vapor mixing ratio	kg kg^−1^	—	HYSPLITE model [32]

N: number.

SSA’s unique periodic changes in meteorology play an important role in worsening air quality, especially during the Harmattan season. Thus, we also considered several temporal predictor variables to investigate the role of meteorology on NO and NO_2_. We measured weather parameters at several sites using Kestrel 5500 (Nelsen-Kellerman, Pennsylvania, USA) and computed the averaged mean temperature, RH, and wind speed for each measurement week. But the weekly averaged values showed minimal spatial variations across sites, thus, we relied solely on weather data from a fixed background site as a representative site. Using the Global Data Assimilation System from the National Oceanic and Atmospheric Administration (NOAA) [32], we computed averaged median mixing layer depth, median incident solar radiation, and mean water vapor mixing ratio at the fixed background site for each measurement week. Daily rainfall data at the Kotoka international airport were used to calculate the number of days it rained in each measurement week.

### 2.3. Model development

Most previous land use regression models relied solely on spatial predictors and could not capture the temporality that is inherent in environmental exposures [33–38]. In this study, we applied mixed effects linear regression models to examine the associations of weekly NO and NO_2_ concentrations with both the spatial and temporal factors [39]. To capture time-dependent variance, we added calendar-month and calendar-week as fixed and random effects, respectively. An indicator for measurement sites was also included as random effects to account for both repeated measurements at the fixed sites and site-specific unmeasured factors.

Like previous studies [40, 41], the weekly ambient NO and NO_2_ concentrations at measurement site *i* on week *j* is assumed to be a linear function specified as:

\begin{equation*}{\text{NO}}{x_{ij}} = {\alpha _0} + {\beta _1}{X_i} + {\beta _2}{\text{Me}}{{\text{t}}_j} + {\beta _3}{\text{Mon}} + {b_i} + {\gamma _j} + {\varepsilon _{ij}}\end{equation*}

where NO*x_ij_* is the concentration of NO or NO_2_ measured at location *i* in week *j; α*_0_ is the fixed intercepts, *β_1_, β_2_*, and *β_3_* are the regression coefficients; *X_i_* is a vector of individual spatial predictor variables assembled in table 1 at site *i*; Met*_j_* is the meteorology data in week *j*; Mon is the calendar month for week *j; b_i_* and *γ_j_* are the random intercepts of site and week; and ${\varepsilon _{ij}}$ is the error term.

#### 2.3.1. Model selection

Our model selection process was aimed at finding parsimonious and generalizable set of predictors with maximum predictive accuracy. We first conducted univariate analysis for all the predictor variables (figures S1 and S2). For each spatial variable, we selected the buffer size with the highest correlation (Pearson *r*) with NO and NO_2_ (figures S3 and S4). We then used a supervised stepwise forward regression selection approach to determine the optimal models. The predictors with the highest adjusted *R* ^2^ were added sequentially to the model and retained if our *a priori* direction of association was confirmed and there was at least 1% gain in the adjusted *R* ^2^ (table S1) [35, 42]. Finally, we checked collinearity; variables with variance inflation factor (VIF) > 3 were removed and the model was rerun. All analyses and model development were implemented with the open-source statistical package R version 4.1.2 (R Project for Statistical Computing). R package ‘lme4’ was used to fit the mixed effects models.

#### 2.3.2. Model validation

A commonly used technique in statistics (or machine learning) to assess the performance and generalizability of a newly developed predictive models is to examine how well the model will perform when predicting at unseen location [43, 44] (i.e. locations in the GAMA other than the 134 measurement sites). Thus, the fit and external predictive power of our final models were evaluated using 10-fold CV [40–42, 45–47]. First, all the samples were randomly allocated into 10 subsets, each containing 10% of the data. Subsequently, by holding out a 10%, the remaining 90% was used to train the model and predict the 10% hold-out data. The process was repeated so that every group was used one time in the validation process. For each iteration, we evaluated model performances by computing the mean absolute error (MAE), root-mean-square error (RMSE) as well as *R*-square (*R* ^2^) between the predicted and the measured values. Our final NO and NO_2_ models are summarized in table 2, and their performances in table 3.

**Table 2.** Associations of measured NO_2_ and NO concentrations with spatial and temporal predictor variables in the final linear mixed models.

NO_2_			NO		
Predictor variables	Buffer size (m)	Coefficient (Std. error)	Predictor variables	Buffer size (m)	Coefficient (Std. error)
Intercept	—	40.1 (5.9)	Intercept	—	61.8 (5.4)
Length of major road^a^	100	5.6 (2.8)	Length of major road^a^	100	23.4 (3.5)
Length of secondary road^a^	200	10.4 (2.4)	Length of secondary road^a^	50	15.8 (2.7)
NDVI^a^	50	–13.7 (1.6)	Presence of bar^a^	500	3.3 (1.8)
Mean wind speed in calendar week^a^	—	–11.0 (1.7)	Mean solar radiation in calendar week^a^	—	−4.0 (2.1)
Mean RH in a calendar week^a^	—	–4.1 (1.4)			
Calendar month			Calendar month		
July 2019	—	28.9 (7.3)	July 2019	0	0
August 2019	—	27.7 (7.7)	August 2019	—	–4.4 (6.0)
September 2019	—	22.2 (7.3)	September 2019	—	9.3 (7.4)
October 2019	—	13.4 (6.9)	October 2019	—	–1.5 (6.4)
November 2019	—	17.7 (6.8)	November 2019	—	–9.1 (7.2)
December 2019	—	24.4 (8.5)	December 2019	—	–12.9 (8.9)
January 2020	—	20.1 (11.3)	January 2020	—	–26.7 (13.2)
February 2020	—	26.9 (7.0)	February 2020	—	–4.0 (7.7)
March 2020	—	14.5 (7.1)	March 2020	—	–12.9 (7.0)
April 2020	0	0	April 2020	—	–35.3 (11.1)
May 2020	—	12.0 (7.6)	May 2020	—	–3.5 (7.1)
June 2020	—	17.9 (8.9)	June 2020	—	0.3 (8.8)

^a^ Standardized: Continuous variables were standardized by subtracting the mean and dividing by the standard deviation. A 1-point change in a standardized variable corresponds to a 1 standard deviation increase on the original scale.

**Table 3.** Model fit and 10-fold cross validation between the predicted and the measured samples.

	Model *R* ^2^				
Model	Fixed	Mixed	RMSE (*µ*g m^−3^)	MAE (*µ*g m^−3^)	*R* ^2^_CV_ (%)
NO	0.66	0.79	21.7	14.9	0.78
NO_2_	0.62	0.88	14.6	10.8	0.80

Model *R* ^2^s fixed effects (spatial-invariant) and random effects (time-varying) variables in the mixed effects regression. We estimated RMSE as ${\text{RMSE}} = \sqrt {\frac{{\sum\nolimits_{i = 1}^n{{\left( {{y_i} - {x_i}} \right)}^2}}}{n}} $, where ${y_i}$ is the predicted value, ${x_i}$ is the observed value; $n$ is the total number of data points; and MAE as ${\text{MAE}} = { }\frac{{{{\mathop \sum \nolimits}}_{i = 1}^n\left| {{y_i} - {x_i}} \right|}}{n}$, where ${y_i}$ is the predicted value, ${x_i}$ is the observed value; $n$ is the total number of data points. CV. We reported information separately for the fixed only (‘Fixed’) and the combined fixed and random ‘Mixed’ components of the models.

#### 2.3.3. Model prediction, population exposure and socioeconomic inequalities in exposure

The final models were used to predict weekly NO and NO_2_ concentrations at 50 m × 50 m resolution across the entire GAMA, using *st_as_stars()* function in the ‘*stars*’ package in *R*. We then generated the same variables in the final model within each grid. The model was run for each grid for each calendar week. The weekly predictions were then summarized and mapped as annual and season-specific (non-Harmattan vs Harmattan) mean concentrations. We also used the predictions to estimate the share of population in the AMA that were exposed to NO_2_ concentration relative to the World Health Organization (WHO) guidelines. This was done by spatially overlaying the predicted NO_2_ concentration surfaces onto 2010 census EA map and summarizing the predicted NO_2_ by the share of population in each EA. We relied on the 2010 census because Ghana’s 2021 census results were not available at the time of this analysis. Here, we focused on AMA as it is the most urbanized and densely populated and the commercialized hub of the GAMA. We chose NO_2_ for this additional analysis because it is a key marker for traffic-related air pollution in cities, and concerns over its adverse health and environmental impacts have resulted in national regulations and international guidelines to minimize population exposures. Unlike NO_2_, NO does not have regulatory guidance.

Similarly, we investigated whether NO_2_ distribution varies by EA level SES in the AMA. Our measure of SES was median household consumption estimated from the 2012 Ghana Living Standard survey combined with the 2010 census, using small area models. Detailed description of how the area-level SES was calculated can be found elsewhere [48]. The EAs were divided into SES quintiles (i.e. 20% of EAs in each group) to represent low-, medium-low-, medium-, median-high-, and high-SES groups. The median NO_2_ levels across the different SES groups were then compared. We also conducted t-test to assess the mean difference in the averaged NO_2_ concentrations between the highest vs lowest SES groups, using a *p*-value cut-off of <0.05.

## 3. Results

### 3.1. Final models and their performance

**Figure 2.**
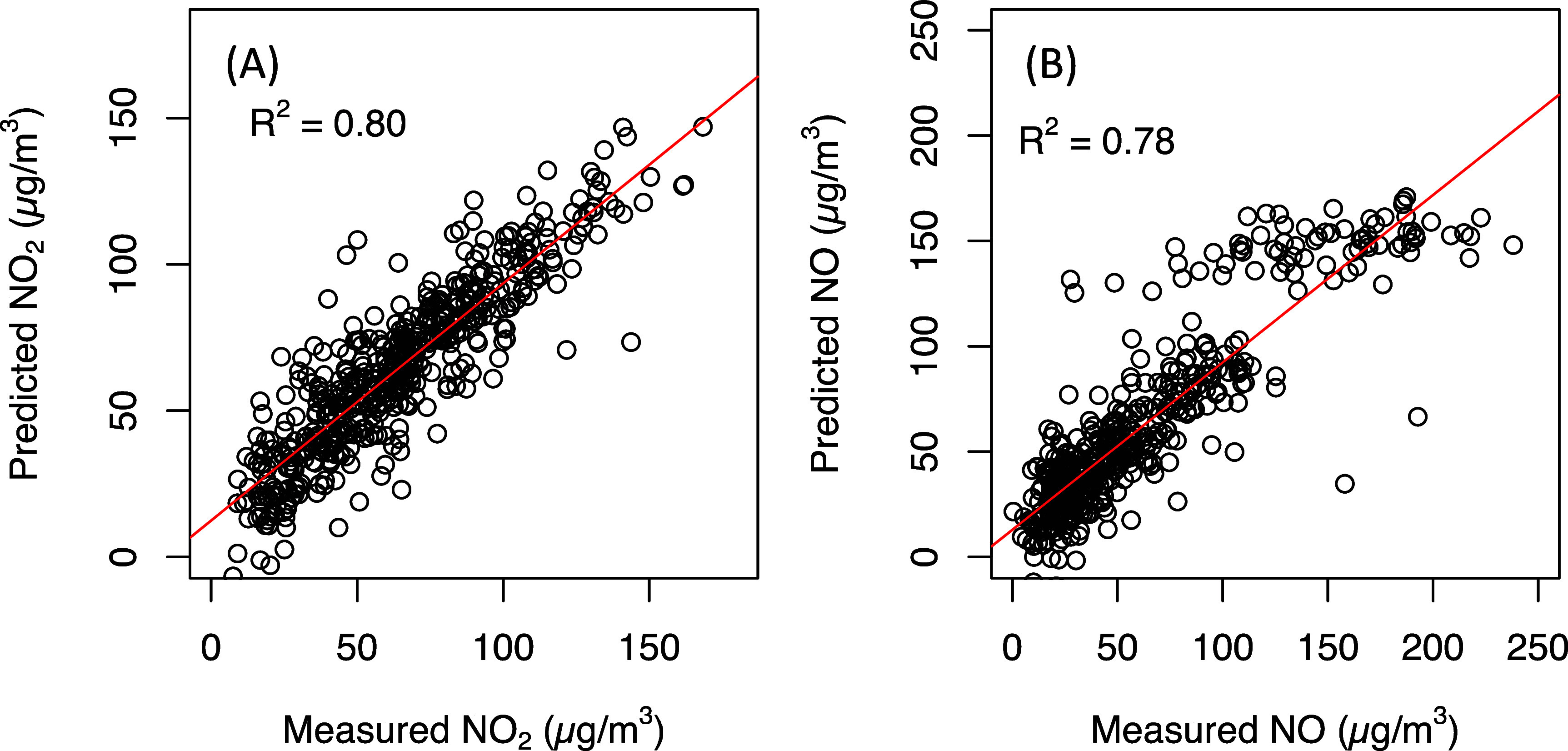
Scatter plots of the measured vs. predicted (A) NO_2_ and (B) NO concentrations based on 10-fold cross-validation.

Table 2 summarizes the final NO and NO_2_ models. The NO model included length of major (within 100 m) and secondary (within 50 m) roads, presence of bars (within 500 m), and mean solar radiation in a calendar week, which explained 79% of the variability in measured NO (*R* ^2^ = 0.79). The NO_2_ model included length of major (within 100 m) and secondary (within 200 m) roads, NDVI (within 50 m), mean wind speed and RH in a calendar week, explaining 88% of variability in NO_2_ (*R* ^2^ = 0.88) concentrations. CV results showed strong correlation between the predicted and the measured NO (*R* ^2^_CV_ = 0.78) and NO_2_ (*R* ^2^_CV_ = 0.80) concentrations, respectively (figure 2). Both RMSE and MAE for NO (21.7 and 14.9 *µ*g m^−3^, respectively) and NO_2_ (14.6 and 10.8 *µ*g m^−3^, respectively) were relatively small if compared with the range of measured concentrations. The VIF values for both models were <2, suggesting little collinearity among variables in the final models. Nevertheless, the NO model performed better at concentrations <150 *µ*g m^−3^ than at higher (>150 *µ*g m^−3^) concentrations (figure 2(B)). This could be due to the fewer number of observations with extremely high concentrations in our dataset (figure 2(B)).

### 3.2. Spatial and temporal patterns of NO_2_ and NO concentrations

**Figure 3.**
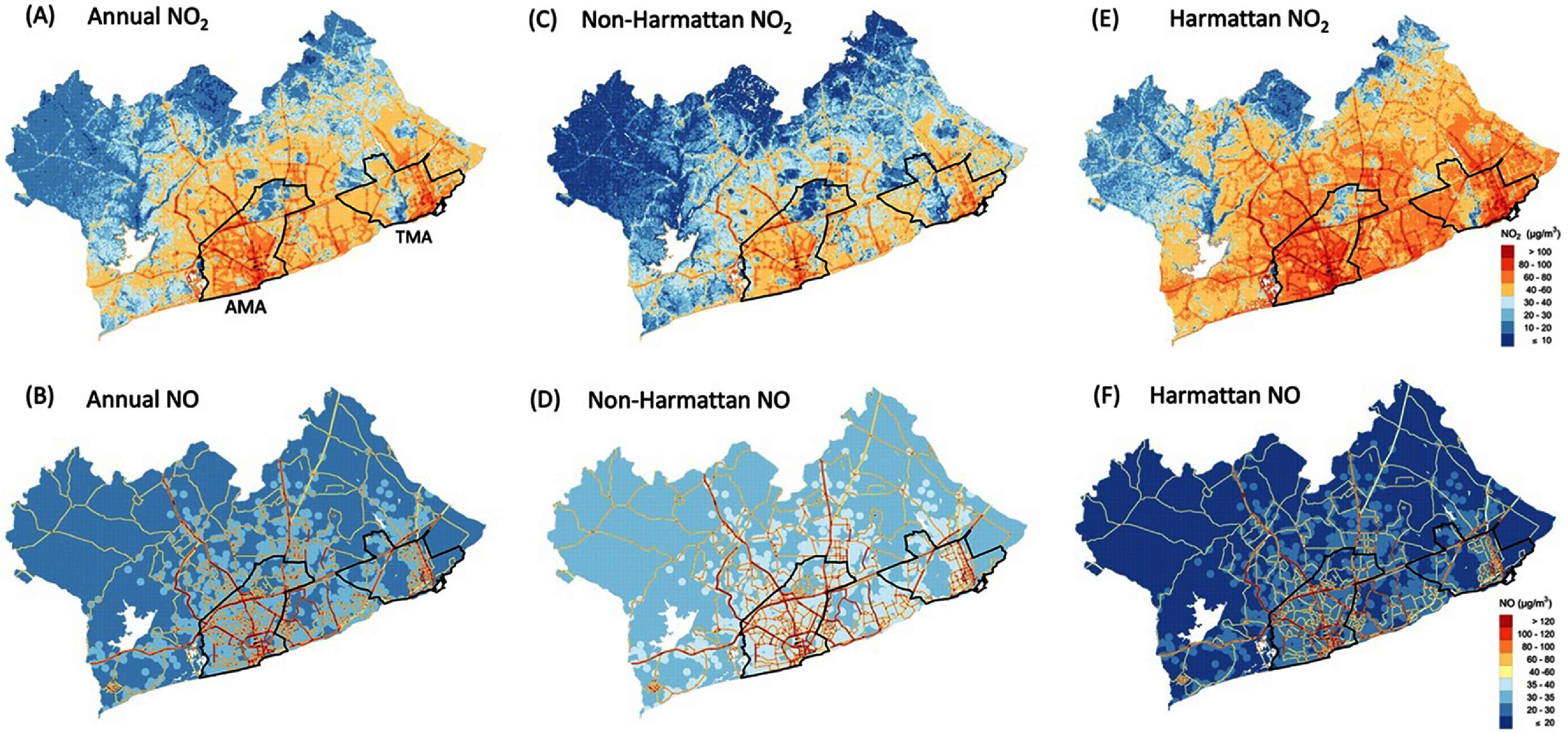
Estimated (A) and (B) annual, non-Harmattan (C) and (D), and Harmattan (E) and (F) NO_2_ and NO concentrations in the GAMA.

**Figure 4.**
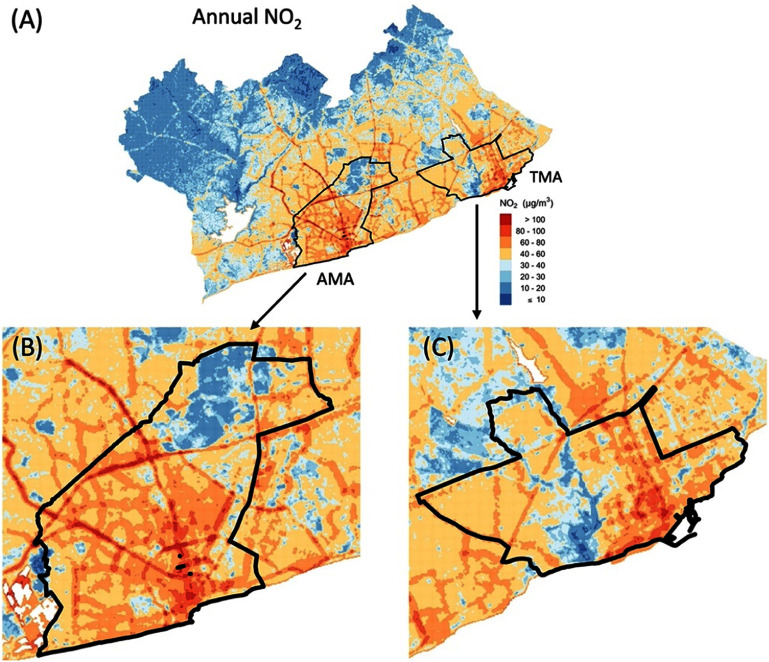
Estimated (A) annual NO_2_ in the GAMA with zoom in for (B) AMA and (C) TMA.

Predicted annual, non-Harmattan, and Harmattan mean NO_2_ and NO concentrations are represented in figure 3, with summary statistics in table 4. The predicted mean (standard deviation, SD) annual NO_2_ concentration for the entire GAMA was 37 (19) *µ*g m^−3^ and ranged from less than 10 *µ*g m^−3^ in the vegetated peri-urban areas to over 180 *µ*g m^−3^ in high traffic areas. The highest NO_2_ levels were concentrated within the city core and along and around major roads in the AMA and TMA (figures 3(A)–(E) and 4(A)–(C)). The mean annual NO_2_ concentration (60 *µ*g m^−3^) in the more congested AMA was nearly doubled that of the entire GAMA. Similarly, the port city district of TMA showed relatively higher NO_2_ concentration compared to the entire GAMA (figure 4(C)). Both AMA and TMA have the highest vehicular traffic congestions in Ghana.

**Table 4.** Predicted NO_2_ and NO concentrations (*µ*g m^−3^) in the GAMA, AMA and TMA.

		Annual		Harmattan		non-Harmattan	
Area	Pollutant	Mean (SD)	Range	Mean (SD)	Range	Mean (SD)	Range
GAMA	NO_2_	37 (19)	1–189	50 (22)	1–195	28 (18)	1–170
	NO	34 (23)	24–514	23 (23)	13–503	41 (23)	31–521
AMA	NO_2_	60 (20)	1.2–179	75 (21)	1.24–195	51 (20)	1–170
	NO	55 (42)	24–514	44 (42)	13–503	62 (42)	31–521
TMA	NO_2_	53 (18)	1–131	68 (20)	1–147	44 (18)	1–122
	NO	43 (30)	24–319	32 (30)	13–308	49 (30)	31–325

Predicted NO concentration across the GAMA showed less spatial heterogeneity but steeper gradient compared with NO_2_. The highest concentrations appeared along road networks, with clusters of relatively high levels in locations with bars (figure 3(B)). These results point to traffic as the most important source of NO emissions in Accra, with additional contributions from commercial biomass and/or generator use. NO is known to oxidize to NO_2_ very quickly, which could explain the steep gradient in NO concentrations away from the major roads. At the same time, this reaction could be responsible for the higher NO_2_ levels in the more urbanized and industrialized areas of the GAMA. Like NO_2_, the annual NO concentrations across the city varied more than one order of magnitude, with overall mean of 34 *µ*g m^−3^ (table 4).

By season, mean NO_2_ concentrations were higher in the Harmattan period than the non-Harmattan, increasing overall by about 80% across GAMA and between 50%–60% in AMA and TMA (table 4). The opposite was true for NO, where the levels during the Harmattan were about 50% lower than in the non-Harmattan.

### 3.3. Population exposure to NO_2_ concentration in the AMA

**Figure 5.**
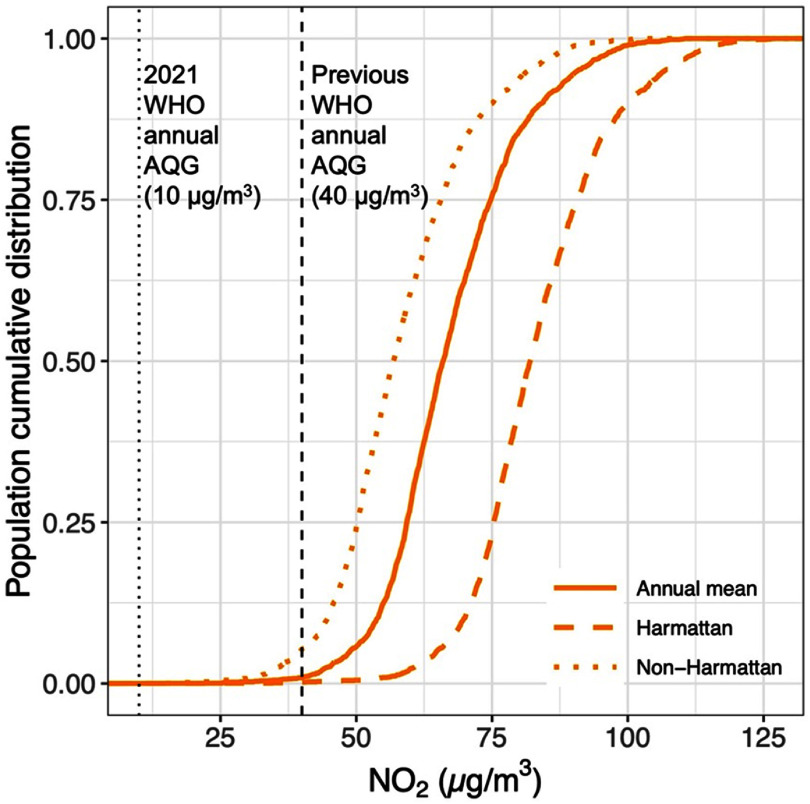
Cumulative densities of the proportion of AMA population living in enumeration areas (EAs) with varying NO_2_ concentration relative to the WHO guideline, by annual, Harmattan, and non-Harmattan averages. The population data used was from the 2010 Ghana Census. The vertical black dash/dotted-lines show the previous (40 *µ*g m^−3^) and the recently revised (10 *µ*g m^−3^) World Health Organization (WHO) annual air quality guideline (AQG) for NO_2_.

The predicted NO_2_ levels for all residents of AMA exceeded the WHO health-based guideline of 10 *µ*g m^−3^, regardless of the season (figure 5). Most of the population in the AMA (80%) lived in areas with annual NO_2_ concentration 5–8 times the recommended guideline (table S2). In the dusty Harmattan season when pollution was highest, over half (56%) of the population lived in areas where NO_2_ concentrations were above 80 *µ*g m^−3^. Though pollution levels improved during the wet non-Harmattan period, still almost 80% of the residents experienced NO_2_ concentrations 4–7 times the recommended guideline.

**Figure 6.**
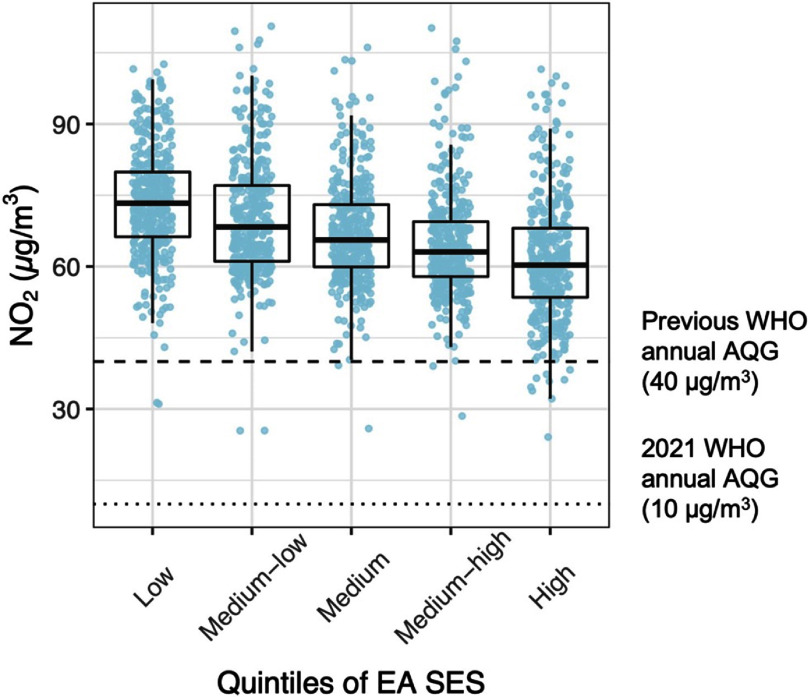
Distribution of enumeration area (EA) annual mean NO_2_ concentrations within quintiles (20% increments) of EA socioeconomic status (SES) in the Accra Metropolitan Area (AMA). SES: EA median log equivalized household consumption. The upper and lower limits of the black box represent the interquartile range of the distribution and the horizontal line within the box represents the median. Each colored point represents an EA average NO_2_ level (*µ*g m^−3^).

In terms of SES, while exposure in both rich and poor communities were above the WHO guideline, there still was a clear gradient in the median NO_2_ concentrations across the EA SES quintiles in the AMA (figure 6 and table S3). The poorest neighborhoods had statistically significantly higher exposure compared the wealthiest (73 vs 60 *µ*g m^−3^; *p* < 0.001).

## 4. Discussion

As SSA rapidly urbanizes, air quality in cities will have major health implications for urban residents. We leverage large-scale measurement data to map out NO and NO_2_ concentrations at 50 m spatial and weekly time resolution over the entire GAMA, one of SSA’s fastest urbanizing metropolises. The final models had high predictive performance and explained much of the variability in the measured NO and NO_2_ concentrations. Road traffic variables were the most important spatial predictors in both models, especially for NO, signifying the role of fresh traffic emissions in the GAMA. This resulted in relatively higher concentrations in the more congested AMA and TMA. We also found a strong negative correlation between greenness NDVI and NO_2_ concentrations in the city, indicating the potential mitigative effect of vegetation in reducing NO_2_ pollution [49]. NDVI in Accra could be closely linked with SES as wealthier communities tend to have more trees than poorer ones. Also, trees/green spaces are known to regulate microclimate by moderating air temperature and humidity, both of which have significant influence on NO_2_ formation and retention. Our finding of the negating role of NDVI points to the need for planting more trees in this sprawling city. Increasing urban green spaces in general can contribute to localized improvements in overall air quality, particularly in areas with high traffic or industrial emissions. For a typical SSA city, residential biomass fuel use could be an important emission source for NO and NO_2_. However, using the 2010 national census data, neither of our final models included household biomass use as an important predictor variable. Interestingly, location of bars (including restaurants) was predictive of NO levels in Accra. This could indicate either commercial biomass use for cooking for sale, the use of disease generators power generation or the presence of cars from customers. Time-varying meteorological variables, including solar radiation (for NO) and wind speed and RH (for NO_2_) were also important predictors. Seasonal changes in these variables produced opposite effects on NO_2_ and NO concentrations in the GAMA. NO_2_ concentrations were higher in the hot, dry and dusty Harmattan period than in the wet/rainy non-Harmattan season. This could potentially be due to more active photochemical and/or aqueous oxidation favored by the meteorological conditions such as stronger solar radiation, and relatively high RH [5], thereby enhancing secondary formation of NO_2_ from NO. Nonetheless, the entire residents of the AMA were exposed to NO_2_ levels exceeding the WHO guideline of 10 *µ*g m^−3^, regardless of the season, with the poorest neighborhoods at much higher risk of exposure than the wealthiest. As Accra expands, there is a need to understand and intervene on factors which drive socioeconomic inequalities in emissions and exposures.

Two studies that empirically mapped NO_2_ levels in SSA were conducted in small urban areas in Ethiopia [20] and Mauritania [21], where the annual mean concentrations were between 5–10 times lower than seen in Accra. To our knowledge, this is the first temporally resolved NO and NO_2_ models developed for a major SSA city, thus we could only compare our models broadly with studies from high-income regions while noting that both the physical and policy environments between the two are completely different. Further, there are limited space-time NO*_x_* models with which to compare our results. Previous studies in high-income country cities have identified traffic as the most important sources of NO*_x_* emissions, just as we found in Accra [34, 50, 51]. While other combustion sources unique to SSA, such as household biomass use and trash burning, could represent non-negligible sources of NO*_x_* emissions, our final models did not include household biomass fuel use as an important predictor. Similar to our results, other studies have also demonstrated a strong influence of climate and photochemistry (e.g. solar radiation, temperature and RH) on NO*_x_* emissions [52]. Our model *R* ^2^s increased by 12% and 5% for NO_2_ and NO, respectively, following the inclusion of meteorological and seasonal variables [40]. Compared to spatial-only models, our space-time models performed similar to some studies in China [36], Europe [34, 53], and South Africa, but better than others [37, 38, 54–56].

Based on the newly revised WHO annual air quality guideline, all residents of AMA were estimated to live in areas where NO_2_ concentrations were far above the recommended health-based annual guideline. Even with the old guideline of 40 *μ*g m^−3^, still the exposure of almost the entire AMA’s population (98%) did not meet the guideline (table S2). Other previous studies have also demonstrated a disproportionate share of poor air quality in low-income neighborhoods when compared to high-income areas [13, 57–60]. We found a similar trend in NO_2_ exposure in Accra as well, with much lower concentrations in the more affluent neighborhoods. This is probably attributed to the higher traffic congestions and emissions in poorer communities and among those who live closer to main roads. Additionally, there could be a higher share of household and commercial biomass fuel use among low-income neighborhoods but our NO_2_ model did not show significant contributions from this source. We acknowledge that the 2010 census biomass data might be outdated, but the overall trend in biomass usage in Ghana has been in decline [61]. Further, substantial disparities in greenspaces between sparsely populated affluent neighborhoods and densely populated poor communities could explain the relatively higher pollution in poorer neighborhoods. Yet, even with such significant disparity in exposure by SES, the median NO_2_ levels in the wealthiest neighborhoods was more than six times higher than the current WHO annual guideline. This calls for a broader policy approach aimed at reducing air pollution emissions across board.

In Accra, concentrations of other pollutants like fine PM_2.5_ and BC also remain detrimentally high [4, 62]. When our results are considered in the context of these other pollutants as well as the increasing urban population growth and economic expansion in the city [4, 62], the data call for an urgent need for equity-focused policy intervention to safeguard the health of Accra residents. These findings further highlight the need to address overall air quality in Accra using an integrated approach with emphasis on equity to reduce the existing within- and between-neighborhood exposure disparities. This will require systematic multisectoral framework that involves aspects related to road traffic emission reduction, environmental management, increasing urban green spaces, improvements to road infrastructure, support for green transportation and cleaner cooking fuels, and enforcement of existing air quality regulations. Our estimates for the non-Harmattan season provide clearer guide for key emission sources that need to be included in any air quality management or policy initiatives for reducing air pollution exposure in Accra and could serve as a roadmap for other cities in the West African region.

### 4.1. Strength and limitations

This is the first fine-scale space-time NO and NO_2_ models developed for a major SSA city, a place where economic growth is making road traffic the dominant source of urban air pollution. We leveraged a large city-wide measurement campaign and provided weekly data over 50 m spatial resolution collected across a calendar year. The data laid the foundation for long-term mapping of inequalities in urban air pollution in a major and growing SSA city and could form the basis for climate and health impact assessments in the SSA context. Further, the data could help track policy interventions designed to improve air quality at the city-scale. Our approach and data sources can be readily replicated in other SSA cities where there is limited long-term city-wide data, especially on combustion related pollutants.

Our study has some limitations. We had no quantitative information on important traffic and other combustion related variables such as road surface material, traffic volume, diesel generator use, informal industries, community biomass use, and trash burning. Some of these data sources are unique to SSA and might improve the model performance if available and may have influenced the variable selection and model performance. Also, the timing of some predictor variables like land-use classification and census population did not align precisely with the timing of the measurement campaign, which may have affected model prediction. Nonetheless, our models performed as well as those conducted in other global studies.

## 5. Conclusion

In addition to PM_2.5_ pollution, gaseous pollutants from combustion sources are rising in growing SSA cities and altering the air pollution mixture. We used large-scale measurement data to map NO and NO_2_ concentrations at fine spatial and temporal resolution in the Accra metropolis. Model predictions show that NO and NO_2_ concentrations are at unhealthy levels in city, with major contributions from road traffic. We also show that while the entire city is severely impacted, residents living in the inner core city, commercial areas, and those in poorer neighborhoods are at the greatest risk of exposure. These results, when combined with the emerging data on fine PM_2.5_, BC, and noise pollution in Accra [4, 5, 48, 62–64] have provided comprehensive information for broader policy intervention and for evaluating the effectiveness of those actions to improve air quality in Accra and elsewhere in SSA.

## Data Availability

The data cannot be made publicly available upon publication because they are not available in a format that is sufficiently accessible or reusable by other researchers. The data that support the findings of this study are available upon reasonable request from the authors.
