# Supplementary material for: Inequalities in urban air pollution in sub-Saharan Africa: an empirical modeling of ambient NO and NO2 concentrations in Accra, Ghana
Source: Environ Res Lett. Author manuscript; Available in PMC 2024 Mar 9. (PMC10897512; doi:10.1088/1748-9326/ad2892)
Supplement: Supplementary data [file EMS194097-supplement-Supplementary_data.pdf]

Supplementary Data

Inequalities in urban air pollution in sub-Saharan Africa: An empirical modelling of ambient NO and NO_2 concentrations in Accra, Ghana

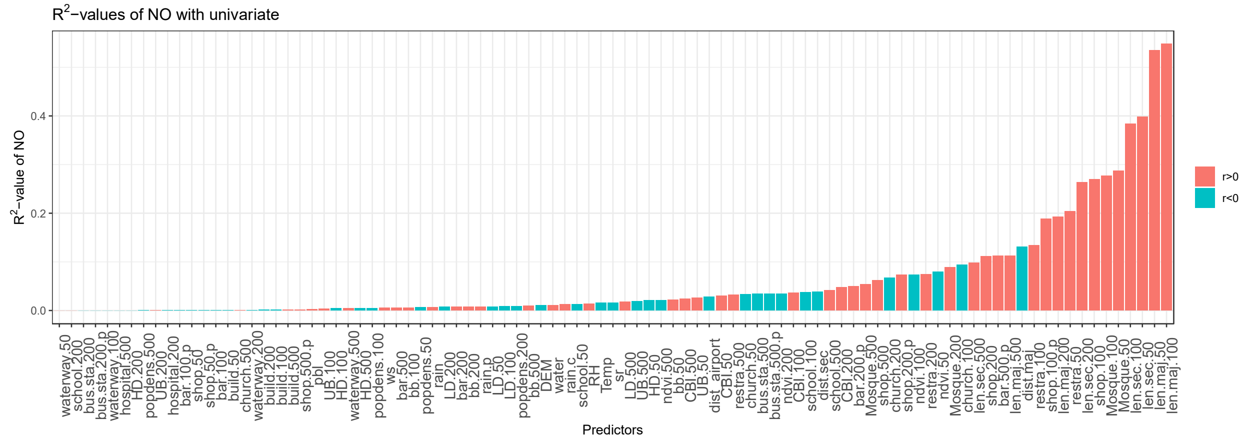

Figure S1. Univariate analysis results of all predictor variables with NO.

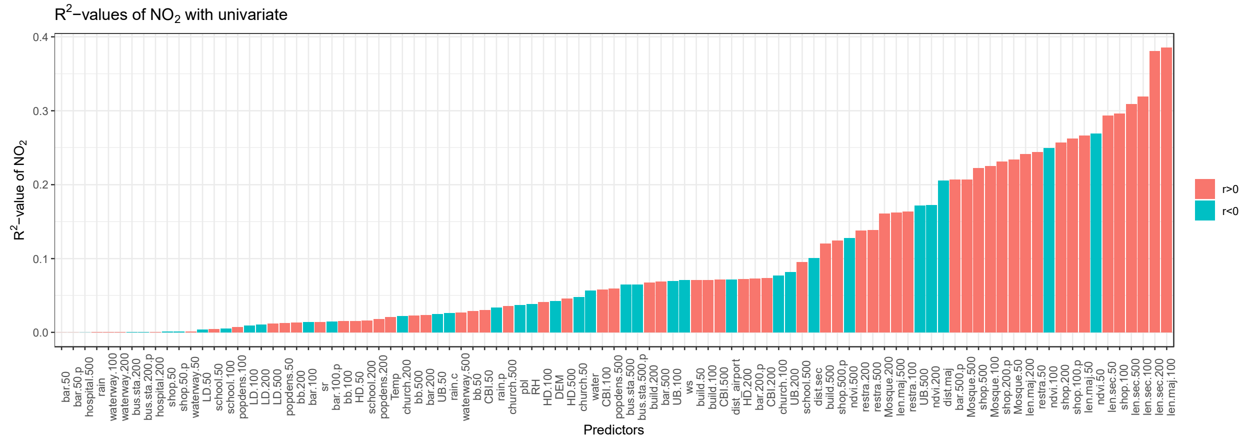

Figure S2. Univariate analysis results of all predictor variables with NO_2.

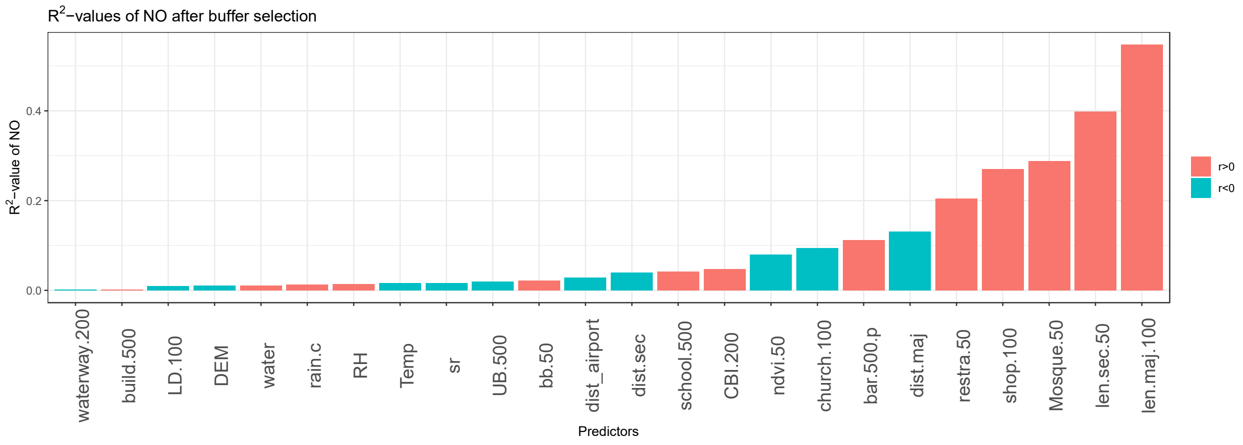

Figure S3. Predictor variables for NO after buffer selection.

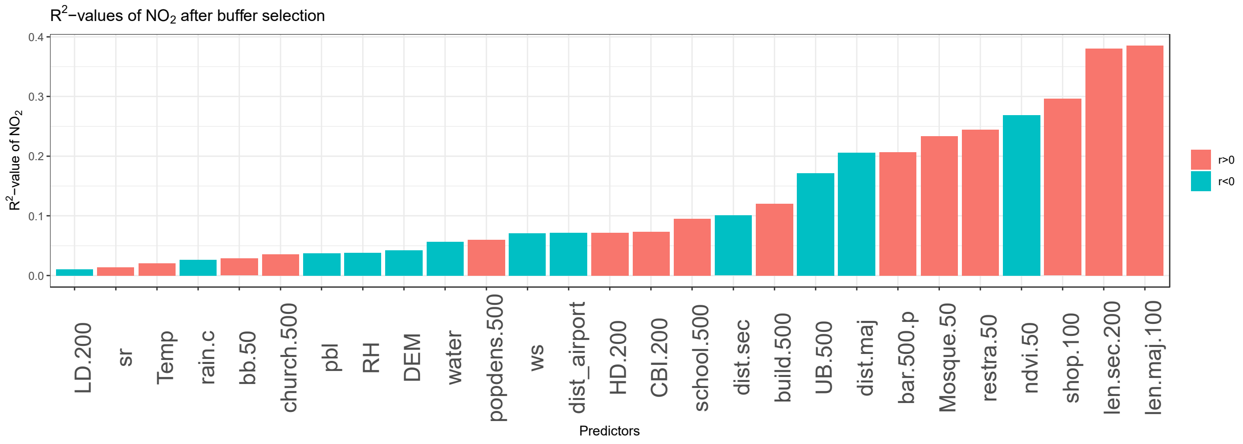

Figure S4. Predictor variables for NO_2 after buffer selection.

Abbreviations: **LD** = low density; **sr** = solar radiation; **Temp** = temperagure; **rain.c** = counts of rained days; **bb.50** = number of biomass burning familiy within 50 meters; **church.500** = church number within 500 meters; **pbl** = planetary boundary layer height; **RH** = relative humidy; **DEM** = digital elevation model; **water** = water vapor mixing ratio; **popdens.500** = population density within 500 meters; **ws** = wind speed; **dist_airport** = distant to the airport; **HD.200** = area of high density within 200 meter; **CBI.200** = area of commercial/business/industrial within 200 meters; **dist.sec** = distant to secondary road; **bar.500.p** = present of bar within 500 meters; **Mosque.50** = Mosque number within 50 meters; **restra.50** = restaurant number within 50 meters; **ndvi.50** = normalized difference vegetation index within 50 meters; **len.sec.200** = length of secondary road within 200 meters; **len.maj.200** = length of major road within 200 meters

Table S1. Supervised stepwise forward selection results of the NO and NO_2 LUR models

NO Model		NO_2 model	
**Predictors**	R^2	**Predictors**	R^2
Total length of major roads (100 m)	0.52	NDVI (50 m)	0.28
Total length of secondary roads (50 m)	0.62	Total length of secondary roads (200 m)	0.44
Calendar month	0.65	wind speed	0.53
Solar radiation in a calendar week	0.66	Total length of major roads (100 m)	0.58
Presence of bar (500 m)	0.66	Relative humidity	0.60
–	–	Calendar month	0.62

Table S2. Estimates of population number and percentage living in enumeration areas with different levels of NO_2 exposure (µg/m^3) in the Accra Metropolitan Area (AMA). Population data is from the 2010 national census.

	Annual		Harmattan		non-Harmattan	
NO_2 (µg/m^3)	**Percentage of the AMA population**	**Number of people in AMA**	**Percentage of the AMA population**	**Number of people in AMA**	**Percentage of the AMA population**	**Number of people in AMA**
< 40	2.2%	36,760	< 1%	10,970	6%	100,360
40-50	4.4%	74,190	1%	19,650	19%	323,150
50-60	22%	368,950	2%	28,609	37%	622,150
60-70	36%	600,740	8%	128,290	23%	387,940
70-80	22%	364,370	3%	551,150	8%	132,830
80-90	8%	132,410	31%	509,950	5%	80,460
90-100	4%	71,470	16%	261,580	< 1%	15,020
>100	< 1%	16,230	9%	154,840	<1 %	3,210

Table S3. Estimated NO2 exposure in the Accra Metropolitan Area (AMA) enumeration area (EA) with different socio-economic status (SES) distribution*. Data summarized as medians and interquartile ranges.

SES	NO_2 (μg/m^3)
High SES EAs	60 (53, 68)
Medium-high SES EAs	63 (58, 69)
Medium SES EAs	66 (60, 73)
Medium-low SES EAs	68 (61, 77)
Low SES EAs	73 (66, 80)

*SES is based on median household consumption (GH₵) within EAs [51]. Quintiles were generated from median EA consumption values within the AMA.
